# Comparative Study of Proximal Tibia and Iliac Crest Bone Graft Donor Sites in Treatment of Orthopaedic Pathologies

**DOI:** 10.5704/MOJ.1707.011

**Published:** 2017-07

**Authors:** ON Salawu, OM Babalola, BA Ahmed, GH Ibraheem, DM Kadir

**Affiliations:** Department of Orthopaedics, Federal Medical Centre, Birnin Kebbi, Nigeria

**Keywords:** bone graft, proximal tibia, iliac crest, bone healing, donor site morbidity

## Abstract

**Introduction:** Bone graft is harvested from bone and used to stimulate bone healing due to its properties. The aims of the study were to compare the quantity of graft harvested from proximal tibia and iliac crest, and the complications at these donor sites.

**Materials and Methods:** This was a prospective study carried out on all consecutive patients who had orthopaedic procedures that required bone grafting at the study centre, from April, 2015 to March, 2016.

**Results:** During the study period, 86 patients were recruited; 43 of these patients had bone graft harvested from the proximal tibia, while the other 43 patients had graft harvested from the iliac crest. There were 53 males, and 33 females. Mean age of patients was 41.2 ± 11 years and 40.8 ± 16 years, for proximal tibia and iliac crest group respectively. Average compressed volume of graft harvested from proximal tibia and iliac crest were 7cm^3^ and 5.5cm^3^ respectively. Non-unions were the commonest indications for bone grafting, femur was the commonest bone requiring bone grafting. Complications such as primary haemorrhage, pain and surgical site infection were commoner with iliac crest than proximal tibia donor sites, however bone graft harvested from both proximal tibia and iliac crest provided adequate bone union of the indicated procedure.

**Conclusion:** Larger quantity of graft can be harvested from proximal tibia than the iliac crest. Though graft harvested from both the proximal tibia and the iliac crest have good healing properties, the proximal tibia donor site gave less complication than the iliac crest.

## Introduction

Bone graft is a piece of bone implanted in between recipient bones so as to promote the healing of the bone as a result of their components physical properties, namely, an osteoconductive matrix, which act as a scaffold to new bone growth; osteoinductive proteins, which stimulate mitogenesis of undifferentiated cells; and osteogenic cells, which become mineralized to form bone at the site where they are deposited^1^.

Bone grafts can be vascularised or non-vascularised. The non-vascularised can further be subdivided into autografts when the graft is taken from the same individual where it is used, allograft when the graft is taken from another individual, usually referred to as cadaveric bone. The autogenous graft can be cortical graft or cancellous graft^1^. Due to the problems associated with autograft, such as donor site complications and inadequate quantity available as often encountered, graft substitutes such as recombinant bone morphogenic protein (rhBMP-2) and ceramic-based bone graft substitutes have been developed and used^1–3^.

Autogenous bone graft is regarded as the gold standard because it is not prone to immunological rejection and it has high osteoconductive, osteoinductive and osteogenic properties compared to other types of grafts and graft substitutes^4–8^. Autografts allow faster time to union, lesser needs for revision procedures, and lower infection rate compared to allograft or recombinant morphogenic protein. The disadvantages of autogenous graft include: increase anaesthesia/surgery time, additional surgical scar, and increased blood loss^9,10^.

Bone grafting is indicated in nonunion, a condition commonly encountered in orthopaedic practice especially in developing countries where traditional bone setters compete with orthopaedic surgeons in managing long bone fractures. Bone grafting is also indicated in joints arthrodesis and filling of bone defects^11,12^.

The reported complications following the use of bone graft are: pain at donor site, infection at the donor site, transmission of infection especially where allograft is used, and fracture at donor site has also been reported^9,10,13–15^. The aims of this study were to compare the quantity of graft harvested from proximal tibia and iliac crest, and also to compare the complications at these bone graft donor sites.

## Materials and Methods

This is a prospective descriptive study, approval for which was obtained from the ethical committee of the study centre. The study spanned a period of one year from April, 2015 to March 2016. All consecutive patients that required bone grafting during the study period were recruited into the study after obtaining consent from them. Proximal tibia graft and iliac crest graft were written on equal number of pieces of papers and each consented patient who required bone graft for his/her procedure was instructed to blindly pick one from these pieces of paper kept in a big brown envelope. The bone graft was taken from either the iliac crest or the proximal tibia of the patients depending on the site on the paper picked by the patient. The quantity of the graft harvested was determined by collecting the harvested cancellous bone graft into the barrel of an empty 10 milliliters syringe.

Intraoperative blood loss from the donor site was estimated using sterile gauze and abdominal packs; mildly soaked gauze = 5mls, moderately soaked = 10mls, and fully soaked gauze = 15mls. While mildly soaked abdominal pack = 50mls, moderately soaked = 100mls, and fully soaked abdominal pack = 150mls. Pain at the graft donor sites were assessed using the numerical rating scale within the 24 hours-postoperative period, and one month and three months post-surgery.

0 = no pain, 1—3 = mild pain, 4—6 = moderate pain, 7—10 = severe pain.

For the proximal tibia bone graft harvest, the patient was placed in supine position following regional or general anaesthesia, with prophylactic antibiotic was given at induction of anaesthesia. The leg was cleaned and draped separately to expose the proximal part of the leg, an incision of about 6 cm was made on the anteromedial surface of the proximal leg between the anterior and medial border of the tibia, about 6 cm below the knee joint line.

The incision was deepened down to the periosteum which was incised and periosteal elevator was used to separate the incised periosteum from the bone. The skin and the periosteum were retracted medially and laterally to expose the proximal tibia. An osteotome was used to create a rectangular cortical window of about 2 cm in width and 3 cm in length between the medial and anterior border of the proximal tibia.

Cancellous bone graft was scooped out from the marrow through the cortical window. The wound was irrigated, the removed cortex at the cortical window site was then reinserted to close the window and skin was closed with 2/o nylon suture and dressing was applied.

For the iliac crest bone graft harvest, the patient was placed in supine position following regional or general anaesthesia, and prophylactic antibiotic was given at induction of anaesthesia. The iliac crest area was cleaned and draped separately. The anterior superior iliac crest was identified, the crest was palpated posterolaterally to get to the iliac tubercle (widest portion of iliac crest). About 6 cm skin incision was made and centered on the iliac tubercle, the skin and subcutaneous layers retracted, and the attached muscles on the lateral aspect of iliac tubercle were detached subperiosteally. Using an osteotome, an opening was created through the lateral aspect of the iliac crest with a medial cortical hinge. Cancellous bone graft was scooped out followed by wound irrigation with saline, the periosteum was apposed with O-vicryl suture, skin closed with 2/O nylon suture and dressing was applied.

The data obtained were recorded in structured proforma which included the biodata, indications for grafting, types of procedure and complications at the graft donor sites. The data generated was analyzed by SPSS version 17. The test of significant association was done by using Chi Square or Fisher exact test as appropriate; the level of statistical significant was set at P < 0.05.

## Results

Eighty-six patients who had procedures requiring cancellous bone grafting were recruited into the study. There were 53 males and 33 females, (F: M = 1: 1.6). The mean age of the patients who had bone graft harvested from the proximal tibia was 41.2 ± 11 years, while the mean age of those who had bone graft harvested from the iliac crest was 40.8 ± 16 years.

Proximal tibia and iliac crest bone graft were taken in 43 patients each, all grafts harvested were cancellous. The average amount of graft taken from the iliac crest was 5.5 cm^3^, while that from the proximal tibia was 7 cm^3^ (χ^2^ = 0.01, P < 0.05). The commonest indication for cancellous bone grafting was nonunion found in 55 (64%) patients, followed by delayed union in 12 (14%) patients, Other indications included graft being used as space filler, in arthrodesis and malunion as shown in Table I. The average time taken for graft harvest from the proximal tibia was 12 minutes while the average time for harvesting bone graft from the iliac crest was 18 minutes (χ^2^ = 8.52, P < 0.05).

**Table I:** Indications for bone grafting

**Indications**		**Number**	**Percentage (%)**
Nonunion			
Femur		22	25.6
Humerus		15	17.4
Tibia		12	14.0
Radius and Ulna		6	7.0
Delayed union			
Tibia		5	5.8
Humerus		4	4.7
Radius and Ulna		3	3.5
Space filler			
Post sequestrectomy		6	7.0
Post curettage benign bone tumor		5	5.8
Malunion			
Tibia		3	3.5
Femur		2	2.3
Radius and Ulna		1	1.2
Arthrodesis			
Ankle		2	2.3
	Total	86	100

Complications observed were primary haemorrhage with an average of about 40 milliliters for proximal tibia graft and about 100 milliliters for iliac crest graft (χ^2^ = 0.03, P > 0.05). Superficial surgical site infections was seen in four patients (4.7%), one patient with proximal tibia graft and three patients with iliac crest graft (χ^2^ = 1.8, Fisher exact P< 0.05). All patients had pain for 24 hours after surgery but with different severity; by one month post-surgery, three of the patients who had proximal tibia graft harvested had mild pain while moderate pain was noted in 12 patients with iliac crest graft, and by three months post-surgery none of the patient with proximal tibia graft had pain at their donor sites, while two patients who had iliac crest graft harvested reported mild pain at their donor sites, as shown in Table II (χ^2^ = 0.06, Fisher exact P < 0.05). All the eighty-six patients had adequate bone union following the various procedures and none of them required re-operation.

**Table II:** Post-operative pain and its severity, using numerical rating scale

**Post-operative pain**	**Number of patients**	**Average severity of pain**
Within 24 hours		
Proximal tibia	43	3
Iliac crest	43	6
One month post-surgery		
Proximal tibia	3	1
Iliac crest	12	4
Three months post-surgery		
Proximal tibia	-	-
Iliac crest	2	2

## Discussion

Bone graft is essential for promoting bone healing in various orthopaedic conditions and diseases. In this study, 86 patients had bone grafting done for various procedures. The graft donor sites in these patients were proximal tibia and iliac crest, 43 each. There were more males than females, (F:M = 1:1.6). This may be due to the fact that males are more prone to injury than females due to the types of activities they are engaged in.

The mean age of patients involved was 41.6 ± 16 years. This means that majority of the patients requiring bone grafting procedures to assist fracture healing were young adults who constitute the work force of the population, conforming the importance of this procedure.

The average volume of graft taken from the iliac crest was less (5.5 cm^3^) than those harvested from the proximal tibia (7cm^3^). This finding was statistically significant (P < 0.05). This means that more graft can be harvested from the proximal tibia than the iliac crest. This finding is contrary to Alt *et al,* who conducted an experimental study on human cadaver and found out that more graft can be harvested from the iliac crest than the proximal tibia^16^. Also Burk *et al* reported that graft harvested from proximal tibia was the least in quantity when compared to anterior and posterior iliac crest^17^. The study by Burk *et al*, harvested corticocancellous graft as against cancellous graft in this study, this may be the reason why the proximal tibia had the least quantity of graft in their study, because the cortical part of graft that can be taken in proximal tibia is less compare to those of the iliac crest.

In our study, the commonest indication for bone grafting was nonunion, 64% n= 55 (Table I). This is due to the high patronage of traditional bonesetters and few orthopaedic surgeons in this part of the country, with the consequence of this is poor fracture management with increased complications. Owoola *et al* also reported nonunion as the commonest indication for grafting in their study^18^. The femur was the commonest bone requiring bone grafting for fracture nonunion, in 22 patients (25.6%). This is because adult femoral fractures must be managed operatively because the surrounding large muscle bulks exert forces that distract the fracture ends and hence prevent healing. Delayed union was the second commonest indication in 12 patients (14%) with the tibia topping the list of bones with delayed union in five patients (5.8%) Other indications for bone grafting were in malunion, arthrodesis and as space filler.

This study showed that it took longer time to harvest bone graft from iliac crest than the proximal tibia, which may be due to the excess subcutaneous fat, the surrounding muscle which need to be dissected and retracted away from the iliac crest before harvesting the graft, unlike the proximal tibia graft site which is subcutaneous. Achieving haemostasis took more time while harvesting graft from the iliac crest than the proximal tibia.

Primary haemorrhage was more at the iliac crest graft site than the proximal tibia site, this is because the iliac crest has surrounding muscles which bleed easily when incised compared to the anteromedial part of the proximal tibia (where bone graft is harvested) that is subcutaneous in nature. However, this incidental finding was not statistically significant (p=0.778).

Superficial surgical site infections occurred in four patients (4.7%) who were known diabetics with controlled blood glucose prior to surgery. One of them had bone graft harvested from the proximal tibia while the remaining three patients had graft harvested from iliac crest. Folds of skin around the iliac crest containing excessive fat may be part of the reasons while infection was commoner at the iliac crest site. *Staphylococcus aureus* was the organism isolated in three patients while *pseudomonas eruginosa* was isolated in one of the patient. The infections were treated with wound dressing and appropriate antibiotics based on sensitivity. All the infections resolved within two weeks of surgery.

Despite the combination of parenteral analgesics (paracetamol, tramadol and diclofenac), all patients had varying degrees of pain within the first 24 hours of surgery. By one month post surgery, three patient who had bone graft harvested from proximal tibia had mild pain, while twelve of those patients with iliac crest site had moderate pain. By the third month post surgery, none of the patient with proximal tibia graft site had pain, while two of those patients with iliac crest graft site still had mild pain. This showed that pain was more intense and lasted longer when bone graft was harvested from the iliac crest than the proximal tibia. This may be due to more soft tissue with nerve endings surrounding the iliac crest graft site than the proximal tibia site (Table II).

Both groups of patients had adequate bone union with bone grafting for the procedures that they used for, none of the procedure requiring re-operation, confirming that the grafts from both sites have good bone healing properties.

Interestingly, none of the patient who had bone graft harvested from the proximal tibia had complain of biomechanical instability of the tibia, this was similar to the findings of Lim *et al* who reported that the tibia is biomechanically stable with full weight bearing ambulation following graft harvest from the proximal tibia^19^. Although the size of the cortical window used by Lim *et al* was smaller than the size used in this study, re-insertion of the osteotomized bony cortex of the cortical window after scooping out the cancellous graft might have contributed to the biomechanical stability of the tibia in this study.

## Conclusion

Harvesting bone graft from the proximal tibia should be considered when carrying out procedures that may require larger amount of cancellous bone for grafting. Even though both proximal tibia and iliac crest grafts have good healing properties, the complications such as post-operative pain, surgical site infection and primary haemorrhage are less when graft is harvested from proximal tibia than the iliac crest.

## References

[1] Nandi SK, Roy S, Mukherjee P, Kundu B, De DK, Basu D (2010). Orthopaedic applications of bone graft & graft substitutes: a review. Indian J Med Res.

[2] Hak DJ (2007). The use of osteoconductive bone graft substitutes in orthopaedic trauma. J Am Acad Orthop Surg.

[3] Bohner M (2000). Calcium orthophosphates in medicine: from ceramics to calcium phosphate cements. Injury.

[4] Pape HC, Evans A, Kobbe P (2010). Autologous bone graft: properties and techniques. J Orthop Trauma.

[5] Flierl MA, Smith WR, Mauffrey C, Irgit K, Williams AE, Ross E (2013). Outcomes and complication rates of different bone grafting modalities in long bone fracture nonunions: a retrospective cohort study in 182 patients. J Orthop Surg Res.

[6] Burchadt H, Enneking WF (1978). Transplantation of bone. Surg Clin North Am.

[7] Samartzis D, Shen FH, Goldberg EJ, An HS (2005). Is autograft the gold standard in achieving radiographic fusion in one level anterior cervical discectomy and fusion with rigid anterior plate fixation?. Spine.

[8] Bauer TW, Muschler GF (2000). Bone graft materials: an overview of the basic science. Clin Orthop Relat Res.

[9] Finkemeier CG (2002). Bone-grafting and bone-graft substitutes. J Bone Joint Surg Am.

[10] Putzier M, Strube P, Funk JF, Gross C, Monig HJ, Perka C (2009). Allogenic versus autologous cancellous bone in lumber segmental spondylodesis: a randomized prospective study. Eur Spine J.

[11] Seiler JG, Johnson J (2000). Iliac crest autogenous bone grafting: donor site complications. J South Orthop Assoc.

[12] Khan SN, Cammisa FP, Sandhu HS, Diwan AD, Girardi FP, Lane JM (2005). The biology of bone grafting. J Am Acad Orthop Surg.

[13] Younger EM, Chapman MW (1989). Morbidity at bone graft donor sites. J Orthop Trauma.

[14] Pollock R, Alcelik I, Bhatia C, Chuter G, Lingutla K, Budithi C (2008). Donor site morbidity following iliac crest bone harvesting for cervical fusion: a comparism between minimally invasive and open techniques. Eur Spine J.

[15] Friedlaender GE (1983). Immune responses to osteochondral allografts. Current knowledge and future directions. Clin Orthop Relat Res.

[16] Alt V, Meeder P, Seligson D, Schad A, Afienza C (2003). The proximal tibia metaphysic: a reliable donor site for grafting?. Clin Orthop Rel Res.

[17] Burk T, Del Valle J, Finn RA, Phillips C (2016). Maximum Quantity of Bone Available for Harvest From the Anterior Iliac Crest, Posterior Iliac Crest, and Proximal Tibia using a Standardized Surgical Approach: A Cadaveric Study. J Oral Maxillofac Surg.

[18] Owoola AM, Odunubi OO, Yinusa W, Unegbu MI (2010). Proximal Tibia Metaphysis: Its Reliability as a Donor Site for Grafting. West Afr J Med.

[19] Lim CT, Ng DQ, Tan KJ, Ramruttun AK, Wang W, Chong DY (2016). A biomechanical study of proximal tibia bone grafting through the lateral approach. Injury.

